# A generalized sense of number

**DOI:** 10.1098/rspb.2014.1791

**Published:** 2014-12-22

**Authors:** Roberto Arrighi, Irene Togoli, David C. Burr

**Affiliations:** 1 Department of Neuroscience, Psychology, Pharmacology and Child Health, University of Florence, via San Salvi 12, Florence 50135, Italy; 2 Institute of Neuroscience CNR, via Moruzzi 1, Pisa 56124, Italy

**Keywords:** numerosity perception, approximate number system, cross-modal perception, reference frame coordinates

## Abstract

Much evidence has accumulated to suggest that many animals, including young human infants, possess an abstract sense of approximate quantity, a *number sense*. Most research has concentrated on apparent numerosity of spatial arrays of dots or other objects, but a truly abstract sense of number should be capable of encoding the numerosity of any set of discrete elements, however displayed and in whatever sensory modality. Here, we use the psychophysical technique of *adaptation* to study the sense of number for serially presented items. We show that numerosity of both auditory and visual sequences is greatly affected by prior adaptation to slow or rapid sequences of events. The adaptation to visual stimuli was spatially selective (in external, not retinal coordinates), pointing to a sensory rather than cognitive process. However, adaptation generalized across modalities, from auditory to visual and vice versa. Adaptation also generalized across *formats*: adapting to sequential streams of flashes affected the perceived numerosity of spatial arrays. All these results point to a perceptual system that transcends vision and audition to encode an abstract sense of number in space and in time.

## 1. Introduction

Animals, including humans, can estimate the approximate quantity of arrays of objects rapidly and relatively accurately, leading to the concept of *number sense* [1,2]. Much evidence suggests that this sense of number is innate. Newborn infants (less than 3 days old) show habituation to number [3], and neurons of the intraparietal sulcus and prefrontal cortex of numerically naive monkeys show selectivity for number [4], suggesting that numerosity is spontaneously represented as a perceptual category within a parietal–frontal network, without need for learning.

However, a truly abstract sense of number should be capable of encoding the numerosity of any set of discrete elements, displayed simultaneously or sequentially, in whatever sensory modality. Some evidence exists for such a generalized number sense. Neurons in the ventral intraparietal sulcus (IPS) and lateral prefontal cortex of behaving monkeys have been reported to encode numerosity for both auditory and visual sensory modalities, suggesting supra-modal numerosity processing [5]. The same group has also described separate populations of neurons in the IPS, responding selectively either to sequential or simultaneous numerical displays [6]. Interestingly, a third set of neurons showed numerosity selectivity irrespective of whether the items were presented simultaneously or sequentially (or both), suggesting that the information converges to a more abstract representation [6]. There is also evidence from functional imaging in humans for a right lateralized fronto-parietal circuit activated by both auditory and visual number sequences [7], and that right IPS is involved in processing both sequential and simultaneous numerosity formats [8].

Psychophysical evidence for a common number sense is somewhat limited. For example, Barth *et al*. [9] showed that there is no measureable cost in reaction times in making cross-format judgements. However, Tokita & Ishiguchi [10] reported that precision in approximate numerosity comparisons between simultaneous, sequential and cross-format presentations are significantly different (lower Weber fractions for simultaneous presentation), suggesting multiple (not unique) mechanisms for numerosity perception in different formats.

As one of the more powerful psychophysical tools for investigating underlying perceptual mechanisms is adaptation [11–13], recently applied successfully to studying numerosity [14,15], we decided to use adaptation techniques to search for a generalized sense of number.

## 2. Material and methods

All visual stimuli were presented on a Nokia 920 C monitor (screen resolution of 800 × 600 pixels, 32 bit colour depth, refresh rate 100 Hz and mean luminance 90 cd m^−2^), subtending 36.5° × 27° at the subjects view distance of 57 cm. Stimuli were created with Psychophysics toolbox (v. 3) for MATLAB [16,17] on a PC computer running Windows 7. Auditory stimuli were digitized at a rate of 65 kHz and presented through two high-quality loudspeakers (Creative SBS 250) flanking the computer screen and lying in the same plane 60 cm from the subject. Speaker separation was around 40 cm and stimuli intensity was 77 dB at the sound source. For the sequential studies, adaptation stimuli were pseudo-random sequences of flashes or tones, displayed for 40 ms (four frames) at an average frequency of 2 or 8 items s^−1^ (see examples in the electronic supplementary material, movies S1 and S2). For example, a 2 item s^−1^ adaptor within a 40 s adaptation period comprised 80 pulses positioned randomly throughout the interval, with the only constraint that two pulses could not fall within 40 ms of each other. Top-up periods of adaptation were presented for 6 s before each trial. Test stimuli were similar, presented pseudo-randomly within a 2 s interval. Visual stimuli were sharp-edged white discs of 90 cd m^−2^ and 4° diameter, displayed 12° to the left or right of fixation (usually in screen centre). Auditory stimuli 500 Hz, ramped on and off with 5 ms raised-cosine ramps.

**Figure 1.**
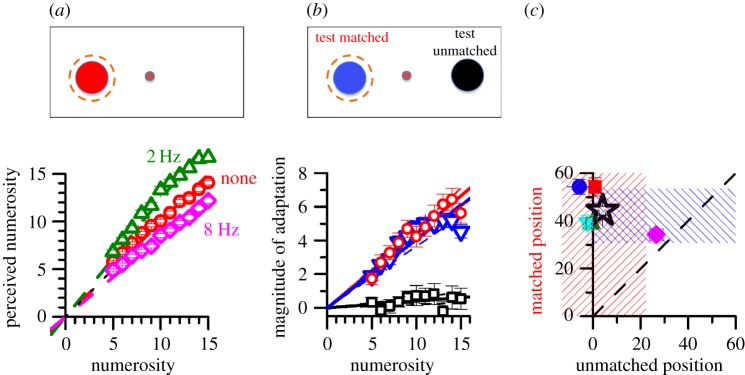
Adaptation to sequential stimuli. (*a*) Perceived numerosity (averaged over trials and subjects) as a function of physical numerosity for the three adaptation conditions, with best-fitting linear regressions (*R*^2^ > 0.98 in all cases). Regression slopes: no adaptation (red symbols) 0.99; 2 flashes s^−1^ (green) 1.23; 8 flashes s^−1^ (magenta) 0.83. Only the curves for 2 and 8 flashes s^−1^ were significantly different from 1 (*p* < 0.001 in both cases). (*b*) Adaptation magnitude: perceived numerosity after adaptation to 2 Hz minus that after adaptation to 8 Hz, as a function of physical numerosity. Blue symbols are taken from the data of figure 1*a* (when testing was predictably on the same side as the adaptor). Red symbols show data when adaptor and test on the same side, black when on opposite sides, both conditions randomly intermingled. All curves are well fitted by linear regression (anchored at zero) to yield an *AI*, an estimate of the magnitude of adaptation. (*c*) AIs calculated for individual subjects for the matched condition plotted against the unmatched condition (data intermingled). The star shows the indexes calculated for pooled data. All except one subject show a clear adaption effect when matched in position, but none when adaptor and test were not matched.

Most data were collected with the technique of magnitude estimation: subjects judged the number of items (visual or auditory, in space or in time) and recorded the perceived numerosity on a numeric pad. Test numerosity ranged from 2 to 20, but we analysed only the range 5–15. This avoided the subitizing range, and also edge effects that may arise (for example from subjects knowing or guessing that the numerosity was never higher than 20). However, analysis of the entire range test gave substantially the same results. Subjects were familiarized with the task with 20 trials, without adaptation, during which correct feedback was given, but no feedback on any other occasion. In the estimation task, the adaptor was generally displayed to the left, followed 900 ms later by a test stimulus of same size, either in the same spatial location as the adaptor or the same eccentricity on the opposite side (insets of figure 1*b*).

We also measured adaptation using a forced-choice paradigm. Here, test and probe stimuli were presented successively after adaptation, first the *test* to the left (same position as the adaptor), then (900 ms later) the *probe* to the right (same eccentricity): subjects judged whether the test or probe appeared more numerous. The magnitude of the standard was chosen at random (between 2 and 20), and the test chosen to differ by a random amount (range ± 7), capped between 2 and 20. As before, adaptation was to the left, first for 40 s then for 6 s top-up periods. After we verified that the adaptation effects were proportional to the magnitude of the stimulus, we plotted the psychometric function as a function of proportional difference between standard and test (difference between standard and test, normalized by the sum of the two). This procedure gave similar results for stimuli in the low (less than 10) and high (more than 10) range.

To study retinotopic/spatiotopic selectivity (figure 3), we used two fixation points: F_1_ 6° left of screen centre and F_2_ 6° to the right. The test was always displayed 6° to the left of F_2_, at screen centre. The adaptor was in the same screen position as the test for the spatiotopic condition, but 6° left of F_1_ for the retinotopic condition. For the ‘full’ adaptation condition, subjects maintained fixation at F_1_ and both adaptor and test were 6° to the right.

In the first cross-format experiment (figure 5), adapters were alternating black and white flash sequences centred 12° in left periphery and test stimuli arrays of 0.4° dots (50% white, 50% black) within a virtual annulus abutting the region of the adaptor flashes (4° and 7° inner and outer diameters). In the other cross-format condition, subjects adapted to an array of slowly moving (0.1° s^−1^) black and white dots (6 or 60 in separate sessions) displayed centrally within a centred 22° diameter region. Dot size was scaled to keep constant (at 10%) the amount of covered area within the adaptation area constant. The test was a sequence of white and black abutting annuli (diameters 11° and 14°).

**Figure 2.**
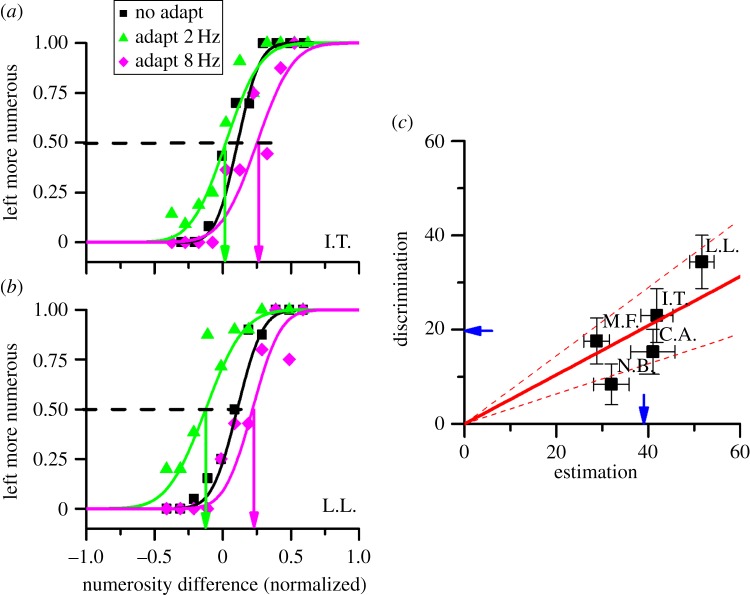
Forced-choice measurement of adaptation to sequential stimuli. (*a*) Psychophysical functions for two example subjects, after adaptation to 2 Hz (green), 8 Hz (purple) or no adaptation (black). The curves plot proportion of trials when the test (falling on the adapted position) was seen as more numerous than the neutral probe, as a function of difference in normalized numerosity (normalized by the sum of test and probe numerosity). Adaptation to 2 Hz shifts the curve leftwards as subjects were biased to perceive the test stimulus as more numerous that it was, and adaptation to 8 Hz shifts the psychometric function rightwards. The point where the best-fitting curves pass 50% is considered the point of subjective equality (indicated by the coloured arrows). (*b*) Adaptation effect from the forced-choice comparison (difference in point of subjective equality of the 2 and 8 Hz conditions) plotted as a function of adaptation effect calculated from the naming experiment. All points are significantly different from 0, in both measures (*p* < 0.05, bootstrap signed test). The red line shows the best-fitting zero-anchored linear regression: its slope of 0.52 suggests that the adaptation estimates from forced choice were on average one-half of those from the naming experiment. The dashed lines indicate 95% confidence interval, and the arrows near the axes the group averages.

**Figure 3.**
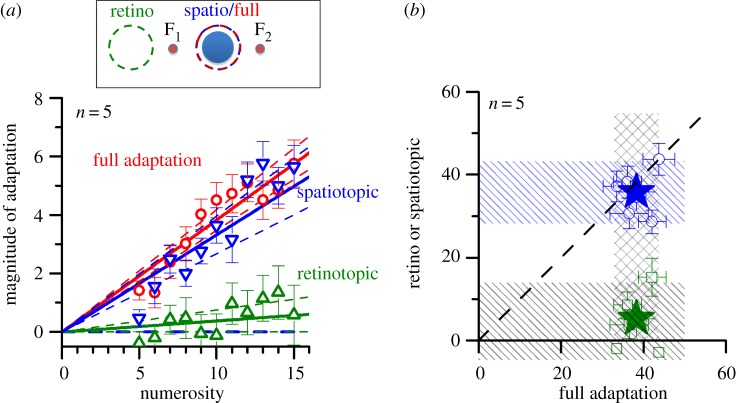
Spatiotopic and retinotopic adaptation. (*a*) Adaptation magnitude measured after a 12° saccade (from F_1_ to F_2_—see inset) between adaptation and test, with the adaptor in the same spatiotopic (blue symbols) or retinotopic (green symbols) position, or both (red symbols). The spatiotopic adaptation was as strong as full adaptation (no saccade), while retinotopic adaptation caused little effect. (*b*) Individual AIs after adaptation in the same retinotopic (green symbols) or spatiotopic (blue) positions, plotted against AIs for the ‘full adaptation’ condition (no saccade between adaptor and test). The dashed lines indicate 95% confidence intervals for all conditions.

**Figure 4.**
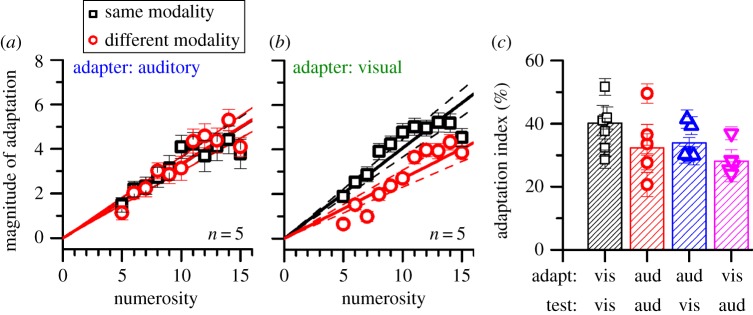
Auditory and cross-modal adaptation. (*a*) Adaptation magnitude after adapting to auditory adaptors and testing with auditory stimuli (black symbols) and visual stimuli (red symbols). (*b*) Adaptation magnitude after adapting to visual adaptors and testing with auditory stimuli (red symbols) and visual stimuli (black symbols). (*c*) Bar graphs summarizing individual AIs (symbols) and pooled data (bars) for all conditions. vis, visual; aud, auditory.

**Figure 5.**
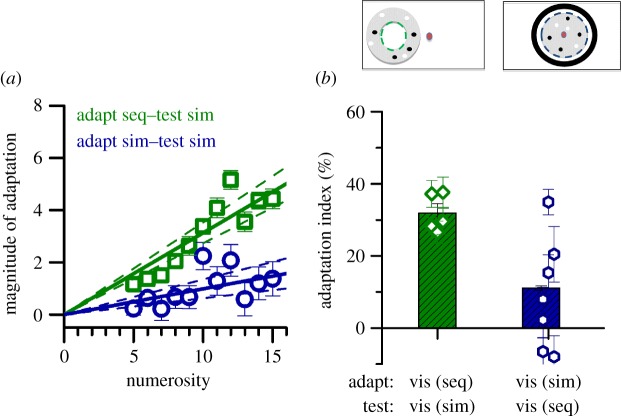
Cross-format adaptation. (*a*) Adaptation magnitude after adapting with peripherally displayed sequential flashes and testing with peripherally displayed spatial arrays of dots (green symbols), or adapting to centrally displayed dot arrays and testing with peripheral flashes (blue symbols). The effect of sequential (seq) on simultaneous (sim) is clear (AI = 0.31), but the inverse was not (AI = 0.10), although both are highly statistically significant (*p* = 0.001). (*b*) Individual AIs (symbols) and pooled data (bars). vis, visual.

A total of eight subjects participated in the study, all naive of the goals of the study, except author I.T., who participated in all experiments. Of the naive subjects, one group of five participated in the experiments shown in figure 1*a*, four of them to the rest of the estimation experiments (figures 1*b*,*c*, 3–5), as well as to the numerosity discrimination experiment (figure 2). Two extra naive subjects were recruited for the cross-format experiment with sequential–simultaneous adaptation to strengthen statistical analyses, given the high variability in this condition). All statistical values refer to Student's *t*-tests.

## 3. Results

We first show that the apparent numerosity of serially presented stimuli is susceptible to adaptation. Observers adapted to pseudo-random sequences of briefly flashed visual stimuli presented to the left visual field (12° eccentricity), for an initial period of 40 s, followed by periods of 6 s re-exposure before each trial. On separate sessions, the adaptors were presented on average at 2 or 8 flashes s^−1^. Subjects then judged the apparent numerosity of test flashes presented to the same spatial region, randomly distributed over a 2 s window (see electronic supplementary material, movies S1 and S2).

Figure 1*a* plots mean estimates of numerosity (averaged over all subjects) as a function of physical number of pulses. The average estimates with no adaptation (red symbols) were quite veridical. The data were well fitted by linear regression anchored at zero (*R*^2^ = 0.99), with best-fitting slope of 0.99. Adaptation to 8 flashes s^−1^ systematically decreased apparent numerosity by 16% at all tested numerosities (slope of linear regression 0.83), and adaption to 2 flashes s^−1^ increased it by 24% (slope of regression 1.23, compared with 0.99 baseline). As the zero-anchored linear regressions all captured more than 98% of the variance in all conditions, it seems that adaptation affected all numerosities by the same proportion.

In order to obtain an index of adaptation, we subtracted the perceived numerosity after adaptation to 2 Hz from that after adaptation to 8 Hz and plotted this difference as a function of physical numerosity (blue symbols of figure 1*b*). This curve is again well fitted by linear regression (*R*^2^ = 0.98) and has a slope of 0.40. We take the slope of this difference curve (multiplied by 100) as the *adaptation index* (AI), an estimate of the magnitude of adaptation.

If adaptation occurs at a perceptual rather than cognitive level (for example, through ‘internal counting’), it should be spatially specific. To test this prediction, we adapted subjects to 2 and 8 flash s^−1^ sequences positioned 12° left of fixation and tested stimuli either in the same or opposite (12° right of fixation: see inset) position, randomly interleaved within sessions. The results are shown in the difference curves (difference in perceived numerosity after adaptation to 2 or 8 Hz) of figure 1*b*, separately for the matched (red symbols) and unmatched conditions (black symbols). Adaptation occurred only when test and adaptor positions were matched: the AI in that condition was 0.44 (*p*_(AI=0)_ < 0.0001), comparable with the first experiment (where the test and adaptor positions always coincided), while the unmatched condition caused almost no adaptation (AI = 0.04). Thus adaptation to sequential number is, like adaptation to simultaneous number, spatially specific.

Figure 1*c* shows the individual data for the matched/un-matched experiment. AIs were calculated in the same way as for group data, separately for the matched position (ordinate) and unmatched position (abscissa). All except one subject showed a clear specificity for position.

The spatial specificity of the adaptation allows us to employ other psychophysical techniques, such as two-alternative forced choice, similar to that used to demonstrate spatial adaptation. Subjects adapted to 2 or 8 flash s^−1^ sequences on the left, then two stimuli were presented sequentially, first a *test* to the left, then a *probe* to the right: subjects reported which appeared more numerous. Average responses of ‘left more numerous' were plotted as a function of the difference between test and probe (normalized to the sum of the two numerosities), to yield psychometric functions like those of figure 2*a*,*b* (two typical subjects). The effect of adaptation is again clear: adapting to 2 Hz shifts the curves to the left (compared with baseline), adapting to 8 Hz to the right. The differences in the points of subjective equality (given by the 50% point of the curves) of the 2 and 8 Hz conditions again gives an index of magnitude of adaptation—in this case 23% and 34% for the two subjects.

Figure 2*c* plots the AIs obtained from psychometric functions against those for magnitude estimation, for each individual subject. The data show that all subjects showed a strong and significant adaptation effect. However, the forced-choice technique tends to give a lower estimate of the adaptation effect, about half that obtained by the naming technique.

We next asked whether the spatial specificity of the adaptation was anchored in retinotopic (eye-centred) or spatiotopic (screen-centred) coordinates. Subjects adapted to 2 or 8 flash s^−1^ sequences while fixating 6° left of screen centre, then saccaded to 6° right of centre before the test sequence was presented. The test was always at screen centre, but in different sessions the adaptor was either in the same spatiotopic (screen) position as the test or the same retinotopic position (left of initial fixation; see inset of figure 3). Figure 3*a* shows the average adaptation effect (difference between 2 and 8 Hz adaptation) as a function of numerosity. When the stimuli coincided on the screen (spatiotopic), the effect was almost as strong as the ‘full adaptation’ condition (when the eyes did not move): AI_full_ = 0.38 (*p* < 0.001) and AI_spatio_ = 0.35 (*p* < 0.001), not significantly different from each other (*p* = 0.18). For the retinotopic condition, however, adaptation was negligible (AI_ret_ = 0.05). Figure 1*b* shows the AIs for individual subjects, plotting both the spatiotopic and retinotopic conditions against full adaptation. All five subjects showed the same effect: strong spatiotopic but little or no retinotopic adaption.

One advantage of serial presentation of items is that it lends itself well to presentation in modalities other than vision (see electronic supplementary material, movies S3–S6, for examples of two cross-modal versions of our task), as it does not require fine spatial resolution. We therefore measured auditory adaptation to sequences of brief tones and tested numerosity estimates of both auditory and visual stimuli. The black symbols of figure 4*a* show that auditory sequences also produce strong adaptation, of the same order as the visual adaption effect (average AI = 0.33). We then adapted subjects to auditory tones and tested with vision (red symbols): adaptation generalizes from audition to vision, with no significant loss in strength (AI = 0.34, *p* = 0.62). Similarly, we measured the effect of adaptation to visual sequences on the perceived numerosity of tone sequences (figure 4*b*, red symbols). Again the adaptation effect was robust, although slightly less than the effect of vision on vision (0.28 cf. 0.40), possibly because audition is a more effective stimulus in time than is vision [18]. Figure 4*c* shows the adaptation effect for the four conditions, both for pooled data (bars) and for individual subjects (symbols).

A crucial test for a generalized number sense is whether adaptation is possible across formats. Subjects adapted to sequences of peripherally displayed flashes (eccentricity 12°) and reported perceived numerosity of spatial arrays of dots of variable numerosity presented around the adaptation location (see inset to figure 5; electronic supplementary material, movie S7). This arrangement of stimuli was devised to optimize adaptation aftereffects as they seemed to be strongest in the periphery. Adaptation to sequential stimuli strongly affected numerosity estimates of simultaneous sequences (figure 5*a*, green data points and lines), with average AIs of 0. 31 (*p* < 0.001), almost as much as for the sequential–sequential adaptation. The inverse condition was to adapt subjects to the numerosity of arrays of dots presented centrally, and test in the periphery, again on the assumption that this should elicit strongest effects. However, adaptation to simultaneous stimuli had little effect on sequential estimates (see the electronic supplementary material, movie S8). The blue symbols in figure 5*a* show the results for central simultaneous adaptors and peripheral sequential tests. Here, the average adaption index was almost three times smaller (AI = 0.10): still statistically greater than zero (*p* = 0.001), but much smaller than the symmetrical condition of sequential adaptation and simultaneous test. We tried other versions of the adaptation test paradigm (including the same set-up as for the sequential–simultaneous adaptation), but none led to significant effects. At this stage, we cannot know whether this difference reflects a real asymmetry, or that we failed to find optimal conditions for this condition.

Figure 5*b* shows the adaptation effect for both cross-format conditions, with bars indicating pooled data while symbols show individual data for all subjects.

## 4. Discussion

The results provide strong support for the existence of perceptual mechanisms that encode numerical quantity from different senses, across space and time. Like most perceptual mechanisms, these are highly susceptible to adaptation. That the adaptation occurs across sensory modalities and across presentation formats shows that these separate ways of representing numeric information are highly interconnected, probably all feeding into one common representation of number. That cross-modal and cross-format adaptation effects were almost as large as within-modal and within-format adaptation suggests that it is the abstract quantity system that adapts, rather than the separate systems that feed it.

It is interesting that the effect of a temporal sequence of items is spatially selective. This is reminiscent of the effect of adaptation on perceived duration: adapting a specific part of the visual field to fast motion decreases perceived duration of grating patches presented to that specific region [19]. Furthermore, adaptation of duration was selective in spatiotopic coordinates, with very little retinotopic adaptation (after compensating for effects on perceived velocity) [20,21]. Similarly, we found that adaptation to sequential number was selective in spatiotopic rather than retinotopic coordinates. This is consistent with the adaptation occurring at moderately high levels of analysis, probably also related to attentional processes [22].

Although adaptation to visual stimuli was highly spatially selective, we found clear cross-modal adaptation with spatially non-localized sounds, generated from a speaker not superimposed on the visual stimuli. Presumably, the auditory stimuli were poorly localizable in space (pure tones generated from a single speaker), and not perceived as conflictual. Under these conditions, visual stimuli dominate auditory stimuli in spatial localization, the well-known *ventriloquist effect* [23,24]. Conceivably, if the sounds were localized more precisely in space, it would be possible to demonstrate spatially selective adaptation. It will also be interesting to study cross-modal numerosity adaptation with tactile stimuli, which are localized spatially better than sounds.

It may be argued that sequential stimuli are not encoded as numerosity *per se*, but as ‘temporal rate’, then multiplied by an estimate of duration. This in itself would be interesting, but unlikely for several reasons. The adaptation we report does not act at low levels of neural analysis (such as primary visual or auditory cortex, selective to temporal frequency), as it occurs cross-modally, to the same extent as within modalities. Also the fact that the selectivity is spatiotopic, rather than retinotopic, points to high-level rather than primary sensory cortex [22,25,26]. But perhaps the strongest evidence against a temporal frequency account is that we find strong cross-format adaptation (from sequential to simultaneous), suggesting that adaptation acts on the abstract *representation* of numerosity, rather than indirectly via temporal rate encoding. Of course, it remains possible that the *mechanisms* that encode sequential number are also involved with estimation of temporal rate, but this would not change any of the arguments advanced here.

Similar arguments have been raised about adaptation to simultaneous representations of numerosity, suggesting that it is texture density, not number, that is being adapted, and that number is perceived only indirectly, via texture mechanisms measuring density [27–29]. Again, this does not seem likely, as much evidence suggests that number is sensed independently of density [30] and that the mechanisms that subserve relatively low (uncrowded) densities are distinct from those detecting higher, ‘crowded’ densities [31]. However, it is difficult to disprove completely the texture-density account with these types of studies. In his critique of the idea that adaptation acts on numerosity, Durgin [28] suggested that ‘cross-modal studies seem a more promising avenue for distinguishing aftereffects of perceived number from retinotopic aftereffects in the early visual analysis of texture density’ (p. R856). We agree completely and believe that our evidence shows unequivocally that adaption can act the abstract *representation* of numerosity, rather than indirectly via texture or other mechanisms.

To conclude, our results fit well with the neurophysiological evidence for distinct neural representation in the intraparietal cortex for representing abstract numerical representations across modalities and formats [5,6], and also in line with psychophysical studies showing that cross-format numerosity judgements have no reaction-time or accuracy cost [9]. Similar results have been reported with monkeys [32]. Taken together, all these studies argue for a generalized sense of number, quite distinct from other visual attributes, such as texture density.

## Supplementary Material

Supplementary materials

## Data accessibility

The datasets with data presented in this article have been uploaded as part of the electronic supplementary material.
