# Supplementary material for: A generalized sense of number
Source: Proc Biol Sci. 2014 Dec 22;281(1797):20141791. doi: 10.1098/rspb.2014.1791 (PMC4240988; doi:10.1098/rspb.2014.1791)
Supplement: Supplementary materials [file rspb20141791supp1.pdf]

Supplementary materials

Movies

1. Example of the serial visual enumeration task, with no adaptor.
2. Example of the serial visual enumeration task, with an adaptor of 2 flashes/sec.
3. Adaptation to serial presentation of 2 flashes/sec, followed by a sequence of a variable number of auditory pulses.
4. Adaptation to serial presentation of 8 flashes/sec, followed by a sequence of a variable number of auditory pulses.
5. Adaptation to serial presentation of 2 tones/sec, followed by a sequence of a variable number of flashes.
6. Adaptation to serial presentation of 8 tones/sec, followed by a sequence of a variable number of flashes.
7. Adaptation to serial presentation of 2 flashes/sec, followed by of simultaneous test presentation of a variable number of dots.
8. Adaption to simultaneous presentation of 60 dots, followed by test of sequential stimuli.

Experimental data

Excel file with all experimental data
